# A novel method-based reinforcement learning with deep temporal difference network for flexible double shop scheduling problem

**DOI:** 10.1038/s41598-024-59414-8

**Published:** 2024-04-20

**Authors:** Xiao Wang, Peisi Zhong, Mei Liu, Chao Zhang, Shihao Yang

**Affiliations:** 1 College of Mechanical and Electronic Engineering, Shandong University of Science and Technology, Qingdao, 266590 China; 2 Advanced Manufacturing Technology Centre, Shandong University of Science and Technology, Qingdao, 266590 China

**Keywords:** Mechanical engineering, Computer science

## Abstract

This paper studies the flexible double shop scheduling problem (FDSSP) that considers simultaneously job shop and assembly shop. It brings about the problem of scheduling association of the related tasks. To this end, a reinforcement learning algorithm with a deep temporal difference network is proposed to minimize the makespan. Firstly, the FDSSP is defined as the mathematical model of the flexible job-shop scheduling problem joined to the assembly constraint level. It is translated into a Markov decision process that directly selects behavioral strategies according to historical machining state data. Secondly, the proposed ten generic state features are input into the deep neural network model to fit the state value function. Similarly, eight simple constructive heuristics are used as candidate actions for scheduling decisions. From the greedy mechanism, optimally combined actions of all machines are obtained for each decision step. Finally, a deep temporal difference reinforcement learning framework is established, and a large number of comparative experiments are designed to analyze the basic performance of this algorithm. The results showed that the proposed algorithm was better than most other methods, which contributed to solving the practical production problem of the manufacturing industry.

## Introduction

With the development of artificial intelligence and big data technologies, intelligent scheduling plays a decision-making role in resource allocation and equipment management of advanced manufacturing systems^1^. Flexible job-shop scheduling problem (FJSP), covering operation research, sequencing theory, and optimization methods, is mainly to determine the processing equipment and process path planning. It is one of the hot topics in the scheduling system^2^. Especially, flexible double shop scheduling problem (FDSSP) that considers job shop and assembly shop is a kind of practical extension of FJSP^3^. Compared to FJSP where assembly-associated jobs can start only after machining is completed, FDSSP is the essential key to collaborating with the scheduling of associated jobs. It can reduce the assembly waiting time of associated jobs when they enter the assembly workshop at the same time as possible, which is conducive to prioritizing the processing of urgent jobs and improving production efficiency.

Over recent decades, FDSSP relatively less studied. It is mainly divided into two categories: non-assembly scheduling (Lu et al.^4^; Thurer et al.^5^) and assembly scheduling (Zou et al.^6^). The former, not involving specific assembly constraints, is to accomplish all tasks of the job shop before assembly operation with only a certain extra assembly period. It is suitable for simple products or short assembly time relative to processing time. The latter should be considered to process and assemble all jobs at the same time. Hence, it has high complexity and practical application.

FDSSP is a comprehensive scheduling problem that offers a subtle fusion of flexible process plans with assembly operations in the two adjacent working shops. It is a hierarchical coupling-constrained optimization problem (HCC). Researchers have spent quite a considerable effort on FJSP, which can be divided into two categories: exact methods (EM) and approximation methods (AM). EM can guarantee the global optimal solution, but usually only solves small-scale problems such as mathematical programming (Zhang and Wang^7^; Nourali et al.^8,9^) and Branch and Bound (B&B) (Brucker and Schlie^10^; Carlos Soto et al.^11^; Özgüven^12^). AM can get the solution to the problem quickly, but it can't guarantee the best explanation. It is very suitable for solving large-scale problems, such as genetic algorithm (GA) (Tian et al.^13^), particle swarm optimization (PSO) (Nouiri et al.^14^), ant colony optimization (ACO) (Huang and Yu^15^; Zhu et al.^16^; Zhang et al.^17^), multi-agent system algorithm (Cheng et al.^18,19^).

These scheduling algorithms are designed along the lines of “modeling, analysis, and optimization”. It cannot effectively use real-time data and historical data, which makes it difficult to deal with the complex and changeable production scheduling problem. However, reinforcement learning (RL) has the advantages of real-time and flexibility. It can directly select behavior strategies according to the input processing state. One of the earliest studies was from Riedmiller and Riedmiller^20^, who proposed a Q-learning algorithm in RL to develop the single-machine scheduling problem to minimize the summed tardiness. The agents used the common scheduling rules as the behavior of the system such as earliest due date (EDD), longest processing time (LPT), minimal slack (MS), etc. Later, some scholars have carried out remarkable in this field, followed by more detail in later sections.

In this paper, the application of DRL, namely deep temporal difference network (DTDN) to scheduling problems in the flexible double shop system is presented. The main contributions of this work are summarized below. (1) The flexible job-shop scheduling model with the assembly constraint level was proposed to redefine the flexible double-shop scheduling problem. Specifically, the jobs assembly constraint level was designed for the assembly shop. (2) We established a deep temporal difference network reinforcement learning framework that defined state space, action space, and rewards space. (3) We applied a deep neural network that inputs the proposed generic state features to fit the state value function.

## Related work

Related work is reviewed under two parts: (1) the flexible double shop scheduling problem and (2) the DRL scheduling.

### Flexible double shop scheduling problem

The flexible job shop scheduling problem, which was introduced by Brucker and Schlie^10^ in 1990, has received widespread attention. However, the flexible double shop scheduling problem has been little studied. Nourali and Imanipour^8^ firstly introduced the assembly job scheduling problem with sequence-dependent setup times. Zhang et al.^7^ deeply investigated distributed particle swarm optimization to solve multi-objective optimization problems (makespan, total tardiness, and total workload). Zheng et al. applied the master-apprentice evolutionary algorithm to cope with the assembly job-shop scheduling problem with random machine breakdown and uncertain processing time. Tian et al.^13^, utilizing a genetic algorithm with variable neighborhood search, studied the distributed assembly job shop scheduling problem to minimize maximum completion time. Cheng et al.^18^ established the adaptive simulated annealing algorithm to solve the mathematical model of the assembly job-shop scheduling with lot streaming. Later, they^19^ discussed the spatial temporal links among three stages with differentiated lot size. Demir and Erden^21^ proposed Genetic Algorithm and Ant Colony Optimization Algorithm to minimize the earliness, tardiness, and due-dates for the dynamic integrated process plan, scheduling, and due date assignment problem. Fan et al.^22^ studied FJSP with lot-streaming and machine reconfigurations (FJSP-LSMR) to minimize the total weighted tardiness. To deal with the two decision steps for FJSP, namely, the job sequencing and the job routing, Zhang et al.^23^ presented a new deep reinforcement learning with multi-agent graphs model. Erden et al.^24^ designed an improved integer and categorical particle swarm optimization algorithm to solve the dynamic integrated process planning, scheduling, and due date assignment problem, in which the earliness, tardiness, and due dates in practical problems are fully considered. Su et al.^25^ established a framework that used the graph neural network and deep reinforcement learning to solve JSP with dynamic events and uncertainty. Fontes et al.^26^ utilized a hybrid particle swarm optimization and simulated annealing algorithm to deal with the JSP with transport resources. Burmeister et al.^27^, applying a multi-objective memetic algorithm with non-dominated sorting genetic algorithm, proposed an energy cost-aware FJSP model based on minimization of both makespan and energy costs. Carlucci et al.^28^ presented a decision scheduling model that simultaneously handled the power constraint and the variable speed of machine tools.

### DRL scheduling problem

In recent years, RL, one of the three types of machine learning, has been successfully applied in some fields such as computing resource scheduling, robot control, and elevator scheduling. Among them, many scholars focused on the production scheduling system by RL. Liu et al.^29^ proposed a parallel algorithm that utilizes asynchronous updates and deep deterministic policy gradients to solve the job shop scheduling problem. Using MMDP to build this model, the state space is represented in the JSSP environment by the processing time matrix, allocation matrix and activation matrix. And, action spaces are denoted by simple scheduling rules. Wei and Zhao^30^ suggested the conception of the production pressure and the job’s estimated mean lateness for respectively defining the system feature and the policy of reward or penalty. The Q-learning algorithm was applied to the determination of the composite machine rules. However, this method can’t describe the actual complex machining process. Luo et al.^31^ used the PPO algorithm to select processes in a discrete action space and verified its superiority in solving flexible job shop scheduling problems. However, the PPO algorithm has not been studied more thoroughly to improve its performance. Mouelhi-Chibani and Pierreval^32^ proposed a neural network (NN) to dynamically select dispatching rules according to the current system status and the workshop parameters. RL can take the scheduling strategy which adapts to the actual system state. Song et al.^33^ presented a method using DRL to learn priority dispatch rules (PDRs) and graph neural networks (GNNs) for FJSP. A new kind of heterogeneous graph scheduling state representation was employed to combine operation selection and machine allocation into one composite decision, which achieved high-quality learning of PDRs. Chen et al.^34^ presented a rule-driven dispatching method based on the data envelopment analysis to solve the multi-objective dynamic job shop scheduling problem. An agent was trained to obtain the elementary rules with the WIP fluctuation of a machine. Shahrabi et al.^35^ introduced the dynamic job shop scheduling problem (DJSSP) that considered machine breakdowns and random job arrivals. In their work, the dispatching rules were based on variable neighborhood search (VNS) and compared with some common dispatching rules and the general variable neighborhood search. Wang^36^ designed an improved Q-learning with the clustering and greedy search policy. A dynamic scheduling system model with multi-agent technology was built including buffer, machine, state, and job agent to maximize the weighted mean of the fuzzy earning. Shiue et al.^37^ established a procedure in which they planned the real-time scheduling knowledge base (RTSKB) using multiple dispatching rules (MDRs). Significantly, MDRs incorporated two mechanisms including an off-online learning module and a Q-learning-based RL module. So far, these algorithms have lacked a unified scheduling problem name. Che et al.^38^, applying a deep reinforcement learning based multi-objective evolutionary algorithm, proposed a multi-objective optimization model for the scheduling problem of oxygen production system. Yuan et al.^39^ suggested a novel framework that translated a combined optimization problem into a multi-stage sequential decision-making problem. This framework is used a multi-agent double Deep Q-network algorithm for FJSP.

This research on the application of RL in these scheduling problems (Table 1) shows that RL is an effective method to solve the scheduling problem. This algorithm has the following characteristics:

1. RL is a decision-making algorithm directly oriented to long-term goals based on state or action value.
2. RL doesn’t need a complete mathematical model of the learning environment. It can imitate human experience, and learn and accumulate experience from the examples or simulation experiments that have been solved.
3. RL needs supervision and teaching. It adjusts the policy according to the evaluation reward obtained in the interaction process. So, it makes optimal responses to different system states.

**Table 1:** Summary of relevant RL methods.

References	Type of problem	Objective	Approach	Dispatching rule and policy
Riedmiller and Riedmiller^20^	Single machine scheduling	Total tardiness	Q-learning	None
Adylin and Oztemel^70^	Single machine scheduling	Mean tardiness	Q-learning	New job insertions
Li et al.^71^	JSP	Long-run average reward	Q-learning	Discrete-event
Chen et al.^34^	FJSP	Makespan	Q-learning	None
Shahrabi et al.^35^	Dynamic JSP	Mean flow time	Q-Learning	variable neighborhood search
Wang^72^	JSP	Earliness Tardiness	Q-Learning	Dynamic greedy search
Shiue et al.^37^	Real-time scheduling	Mean Standard deviation	Q-Learning	Multiple dispatching rules
Liu et al.^29^	JSP	Makespan	DDPG	Multiple dispatching rules
Luo et al.^31^	JSP	TWT Machine utilization rate Machine workload	HMAPPO	Multiple dispatching rules
Song et al.^33^	FJSP	Makespan	PPO	Multiple dispatching rules
Che et al.^38^	FJSP	Total operating cost Switching times	DRL-MOEA	Multiple dispatching rules
Yuan et al.^39^	FJSP	Makespan	MADDQN	Multiple dispatching rules

## Problem formulation

### Mathematical model

**Figure 1:**
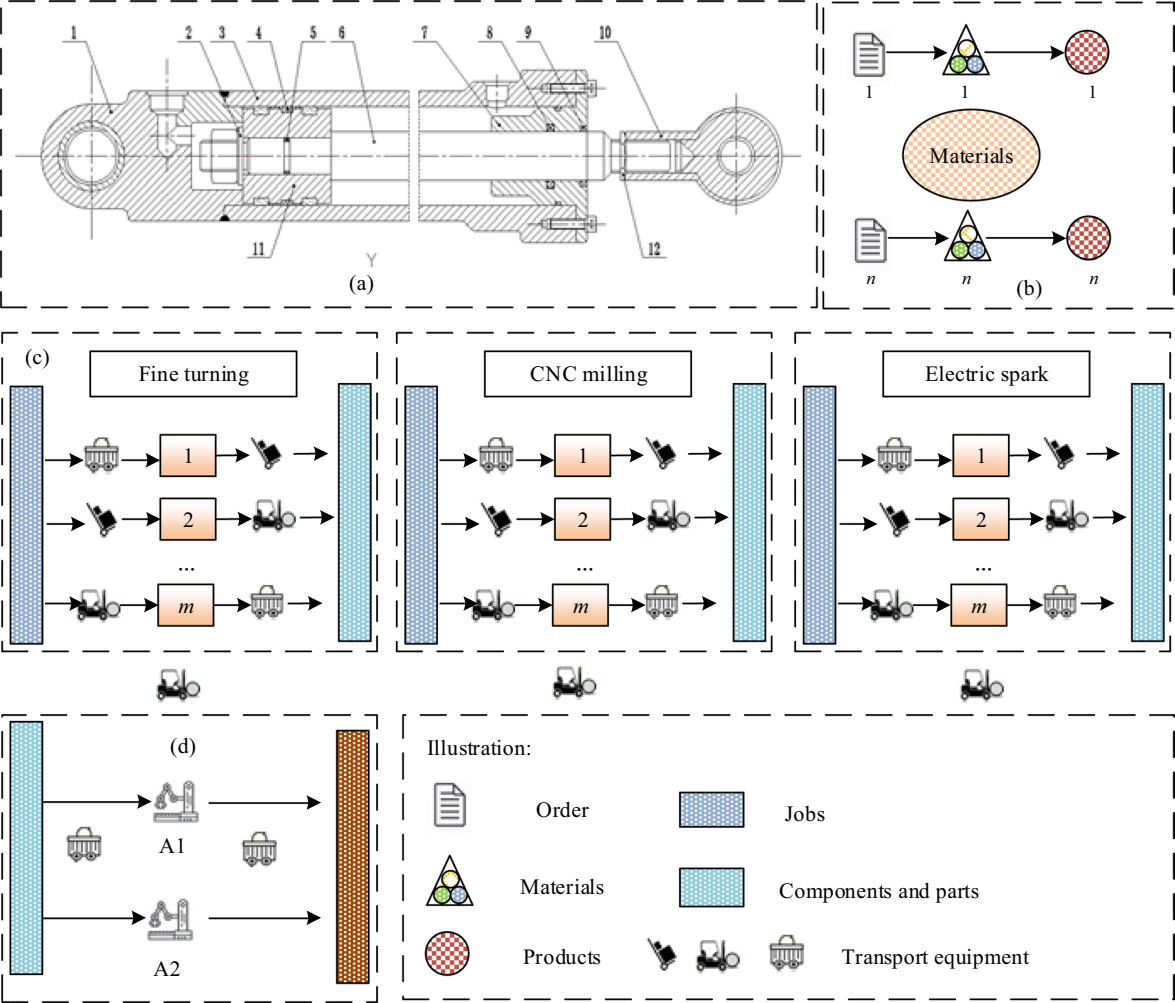
Integrated production of hydraulic cylinder: (**a**) structure charts; (**b**) production layout; (**c**) job shop; (**d**) assembly shop.

We introduce FDSSP by considering the production scheduling problem of the hydraulic cylinder. The hydraulic cylinder processes flow diagram is simplified to a production scheduling model in Fig. 1. Each cylinder ^40,41^ is assembled from several components: body, bottom, piston, piston rod, lifting lug, O-ring, seal ring, piston pin, and wiper, as shown in Fig. 1a. The cylinder body 3 is generally made of seamless steel pipe. Its internal machining accuracy is highly required. Piston 4 and piston rod 6 are connected using snap ring 2. The piston rod 6 is guided by guide sleeve 7 and sealed by seal ring 5. Cylinder bottom 1 and body 3 are respectively opened with oil inlet and outlet ports. When the right chamber of the hydraulic cylinder is filled with oil, the piston moves left. Inversely, the piston moves right.

The shop floor is divided into two areas, namely the job shop and the assembly shop. The product is started from the order and is finished with the assembly (Fig. 1b). The job shop is equipped with three machines (fine turning, CNC milling, and electric spark) (Fig. 1c). The assembly workshop has two assembly robots ($A_{1}$, $A_{2}$) (Fig. 1d). Each operation can be completed by multiple alternative machines. After each operation *k* is completed, job *j* needs to enter the quality control center for quality inspection. If the quality is acceptable, a job is moved to the next operation *k* + 1; if instead, it is returned to the current operation to be queued and reworked again. The assembly operation is a complete kit assembly, which means that the assembly operation does not begin until the all job is completed.

Since the assembly operation may be relatively short and fixed, the planned start time of the assembly operation can be extrapolated from the delivery date of the order. In this paper, we concentrate on the job shop scheduling in a way that the completion time of each job is as close to the planned start time of the assembly as possible. The assembly shop is defined as the assembly constraint level.

Based on the above example, the FDSSP can be described as follows: supposing that there are *n* jobs to be processed in the job shop equipped with $m$ machines. Each job *j* ($j \in \{ 1,2, \ldots ,N\}$) including $O$ operations *k* ($k \in \{ 1,2, \ldots ,O\}$) needs to be processed according to the specified route. Each operation *k* can be selected processing on any powerful machines m ($m \in \{ 1,2, \ldots ,M_{ij} \}$) in $M_{ij}$ machines. Meanwhile, the machine *m* can process different operations *k* of different jobs. Hence, there is a great discrepancy in the processing time of the operation $k$ on different machines*,* which makes the study of scheduling algorithms particularly significant. The model parameters and indices are shown in Table 2.

**Table 2:** Model parameters and indices.

Sets	Description	Indices	Description
*J*	Set of the job	*j*	Indices of the job, $j\in J$
*M*	Set of the machine	*m*	Indices of the machine, $m\in M$
*O*	Set of the operation	*k*	Indices of the operation, $k\in O$

Job-related parameters	Description		
*at*	Arrival time of products		
*d*	Delivery time of products		
*apt*	Assembly time of the products		
$o_{jf}$	The first operation of the process path		
$o_{jl}$	The last operation of the process path		
$t_{jkm}$	Processing time of machine m used in the operation ${k}_{j}$		
$S_{jkm}$	Start time of machine m used in the operation ${k}_{j}$		
$C_{jkm}$	Processing time of machine m used in the operation ${k}_{j}$		
$C_{j}$	Processing time of the last operation for the job $j$		
$C_{\max }$	Makespan of the job $j$(maximum processing time of the last operation for the job $j$)		
*L*	Extreme value		

#### Assembly restraint level definition

The job after assembly is referred to as the constrained job, and the job before assembly is referenced as the front job. Firstly, according to the assembly constraint relationship, all jobs constraint levels that have no tight front constraint are set to 1. Jobs with undefined constraint levels make up the job set, which is denoted by *U*. Then, the job set $J_{set}$ is formed from *U* in sequence taking out all tight front jobs $J_{k}$. Determining whether the constraint levels in ${J}_{set}$ have all been determined. If so, the level of the job $J_{k}$ is set to $\max (L(J_{set} ) + 1)$, i.e., $L(J_{k} ) = \max (L(J_{set} )) + 1$. When not, it puts the job $J_{k}$ back into *U* until the constraint levels of all jobs have been determined.

Other assumptions are considered as follows:

1. The processing times of each operation by each machine are determined and known.
2. Each job can select only one process path. And, one operation can only be processed by one machine at a time.
3. The sum of the start time and processing time of an operation is less than or equal to the makespan of the operation.
4. The makespan of the previous operation is less than or equal to the start time of the next operation.
5. Completion time of products is the sum of processing time and assembly time.
6. The operation of each machine is cyclic.
7. Intermediate conversion time of the job, transferring from the job shop to the assembly shop, is omitted.

Decision variables:

$$x_{jkm} = \left\{ {\begin{array}{*{20}l} {1,} \hfill & {\quad if\;operation\;k\;of\;job\;j\;is\;processed\;on\;machine\;m} \hfill \\ {0,} \hfill & {\quad otherwise} \hfill \\ \end{array} .} \right.$$

According to the literature reviewed^7,42–45^ makespan is the most sufficiently studied objective. In this study, the objective of the model is as follows:

#### Makespan

1 $$\min \left( {C_{\max } } \right) = \min \left( {\mathop {\max }\limits_{1 \le j \le n} \left( {C_{j} } \right)} \right) = \min \left( {\sum\limits_{1}^{n} {x_{jkm} t_{jkm} } } \right).$$

### Transformation of scheduling problem

#### Definition of state-space

The state features can reflect the main features of the production environment. The division of state space is the basis for the reasonable selection of scheduling rules for the system. Nevertheless, owing to the constantly changing production environment, the complete system state is continuous and often described by tens or even dozens of state characteristics on the job shop.

To describe the state space in detail, the following state features are defined:

1. The state features can describe the main features and changes of the scheduling environment in detail, including the global features and local features of the system.
2. The states of all problems are represented by a common feature set.
3. Different scheduling problems can be represented and summarized by state features.
4. State feature is a numerical representation of state variables.
5. The state should be easy to calculate.

To facilitate the expression with the formula, the processing state of the processes is recorded as ${P}_{jk}=\{\mathrm{0,1}\}$, i.e., the operations are not processed is $P_{jk} = 0$, and has been processed is recorded as $P_{jk} = 1$. The operations to be processed on the machine are arranged in descending order of time length, and the resulting process sequence is denoted as $list(m) = \{ J_{m1} ,J_{m2} ,...,J_{{mv_{m} }} \}$, where $v_{m}$ is the number of processes to be processed on machine *m*. As shown in Table 3, we define ten state features of the shop environment.

**Table 3:** State features.

No	State features	Description
1	$x_{m,1} = \sum\nolimits_{{j = 1,k = 1,P_{j,k} = 0}}^{{O_{jp} }} {T_{j,k} }$	Total time of operations to be processed on machine *m*
2	$x_{m,2} = \sum\nolimits_{{j = 1,k = 1,P_{j,k} = 0}}^{{O_{jp} }} {T_{j,k} }$	Total time of operations processed on machine *m*
3	$x_{m,3} = J_{m,1}^{{P_{j,k = 0} }}$	Time of the first operation in the sequence $List(m)$ to be processed on the machine *m*
4	$x_{m,4} = J_{m,2}^{{P_{j,k = 1} }}$	Time of the second operation in the sequence $List(m)$ to be processed on machine* m*
5	$x_{m,5} = W_{j,1}^{{P_{j,k = 1} }}$	Among all future operations, the time of the first operation in the sequence $List(m)$ to be processed on the machine *m*
6	$x_{m,6} = W_{j,2}^{{P_{j,k = 0} }}$	Among all future operations, the time of the second operation in the sequence $List\left(m\right)$ to be processed on the machine *m*
7	$x_{m,7} = \sum\nolimits_{i = 1}^{n} {(1 - P_{j,k} )}$	Total number of operations for all future processes on the machine *m*
8	$x_{m,8} = \sum\nolimits_{i = 1}^{n} {(1 - P_{j,k} )} T_{j,k}$	Total time for all future operations on machine* m*
9	$$x_{m,9} = \left\{ {\begin{array}{*{20}l} {0,} \hfill & {if\;machine\;is\;idle} \hfill \\ {1,} \hfill & {if\;machine\;is\;processing\;a\;job} \hfill \\ \end{array} } \right.$$	Machine states
10	$x_{m,10} = \sum\nolimits_{{J_{k} = 1}}^{n} {(L(J_{k} ))}$	Total number of all jobs assembly constraint levels on the machine* m*

#### Definition of action space

Panwalker and Iskander^46^, summarizing the previous studies, elaborated 113 different combinations of dispatching rules. These rules defined the useful types of problems and measures of performance. The SCH is chosen to define a candidate set of behaviors for each machine, where priority assignment rules for reinforcement learning can overcome short-sighted natures. Behaviors that are relevant or irrelevant to the conversion should be adopted to take full advantage of existing scheduling theory and the ability of the intelligence to learn from it. In Table 4, eight common behaviors are selected as candidate sets.

**Table 4:** Dispatching rules.

No	SCH	Description
1	First come first served (FIFO)	Processing in sequence according to the arrival order of the job
2	Shortest processing time (SPT)	Sorted by the total processing time of the job on all machines from shortest to longest
3	Shortest remaining processing time (SRPT)	Sorted by the remaining processing time of the job on all machines from shortest to longest
4	Most operations remaining (MOR)	Sorted by the number of the remaining operations on all machines from shortest to longest
5	Earliest due date (EDD)	Sorted by the due date from shortest to longest
6	Apparent tardiness cost (ATC)	Sorted by the tardiness cost from shortest to longest
7	Total least operations remaining (TLOPR)	Sorted by assembly-related constraints of the job from shortest to longest
8	Select no job (SNJ)	Machines don’t select processing each job

#### Definition of rewards

The definition of the reward function is closely related to the objective function. The agent is rewarded according to the result of the change of the system state after the implementation of the synthetic behavior and the reward function. The reward function is chosen to be defined according to the following rules.

1. The immediate reward for each state transition reflects the immediate effect of the action performed, which results in a short-term impact on the scheduling plan.
2. The cumulative total reward result reflects the long-term outcome of the execution strategy, denoted as the optimal value of the objective function.
3. This reward function can be applied to scheduling problems of different sizes.

The literature^47^ shows a direct relationship between ${C}_{max}$ and machine utilization (e.g., minimizing the makespan is equal to maximum machine utilization). This study is devoted to addressing minimizing the makespan. The immediate reward earned for each state transition reflects the immediate impact of the action performed. It also represents the short-term impact of the action on the scheduling scheme. Cumulative rewards reflect the long-term effects, which is the goal of RL maximization.

2 $$U_{ave} (t) = \frac{1}{m}\sum\nolimits_{k = 1}^{m} {U_{k} (t) = \frac{1}{m}} \frac{{\sum\nolimits_{1}^{n} {\sum\nolimits_{k = 1}^{{O_{i} (t)}} {t_{jkm} x_{jkm} } } }}{{C_{\max } (t)}}.$$

where $U_{ave} (t)$ is the average machine utilization rate. Let $C_{\max } (t)$ denotes the completion time of the last operation assigned on machine *m* at scheduling point *t*. $O_{t}$ is the current number of operations for the job *i* that have been assigned. Define the machine m utilization rate as $U_{k} (t)$, which can be calculated by $U_{k} (t) = \frac{{\sum\nolimits_{1}^{n} {\sum\nolimits_{k = 1}^{{O_{i} (t)}} {t_{jkm} x_{jkm} } } }}{{C_{\max } (t)}}$. Let $r_{k} = U_{k} (t) - U_{k - 1} (t)$, then the cumulative reward *R* can be calculated as follows:

3 $$R = \sum\limits_{k = 1}^{K} {r_{k} } = \sum\limits_{k = 1}^{K} {\left( {U_{k} (t) - U_{k - 1} (t)} \right)} = U_{k} (t).$$

##### Proof

4 $$\begin{aligned} & \sum\nolimits_{1}^{K} {\left( {U_{k} (t) - U_{k - 1} (t)} \right)} \\ & \quad = U_{1} (t) - U_{0} (t) + U_{2} (t) - U_{1} (t) + \cdots + U_{k} (t) - U_{k - 1} (t) \\ & \quad = U_{k} (t) - U_{0} (t) \\ & \quad = U_{k} (t) = \frac{P}{{C_{\max } (t)}}. \\ \end{aligned}$$

where* k* is the counter for the allocation operation. It can be considered as a discrete-time step in RL. $P = \sum\nolimits_{1}^{n} {\sum\nolimits_{k = 1}^{{o_{i} (t)}} {t_{jkm} x_{jkm} } }$*.* $U_{k} (t)$ and $C_{\max } (t)$ are machine utilization and makespan at time step *k*.

## Proposed methods for the FDSSP

### Related work of RL

RL is a specific class of machine learning (ML) problems that can achieve global optimality^48–50^. In an RL model^51^, the decision-maker chooses an appropriate action by observing the environment and is rewarded for doing so. RL algorithms needn’t know many states and the state transfer probability matrix during iterations. RL is transformed into the model of solving the optimal solution of Markov decision models, which is mainly used to solve sequential decision problems. The most important feature of RL is that there is no correct answer in the learning process, rather learning is done through reward signals.

**Algorithm 1:**
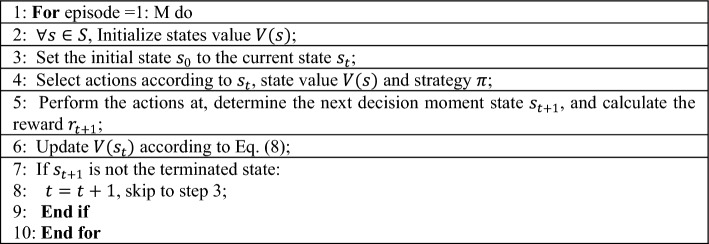
Procedure of TD with evaluating state value

### Markov decision processes (MDP)

Markov is the property that the next state $s_{t + 1}$ in an RL system is related only to the current state $s_{t}$. The Markov decision process is described by 5-tuple as follows:

5 $$E = \{ S,A(s),P,R,\gamma \} .$$

where *S* is a finite set of states, characterizing the description of the environmental state; *A* is a finite set of action spaces, representing the set of behaviors that can be taken; *P* is a state transfer rate function; *R* is a reward function; $\gamma$ is a discount factor.

The objective of RL is to enable the agent to find an optimal strategy $\pi^{ * }$ through continuous experimentation in the environment that maximizes the expected cumulative reward function obtained by following the strategy from any state. The reward function is determined by further defining the value function. The state-value function $v_{\pi } (s)$ and the action-value function $q_{\pi } (s)$ under the strategy are defined as follows.

6 $$\begin{aligned} v_{\pi } (s) & = E\left[ {G_{t} |S_{t} = s} \right] \\ & = E_{\pi } \left[ {\sum\limits_{k = 0}^{\infty } {\gamma^{k} r_{t + k + 1} |S_{t} s} } \right] \\ & = \sum\limits_{a \in A} {\pi (s|a)} q_{\pi } (s,a) \\ & = \sum {\pi (s|a)} \left[ {R_{s}^{a} + r\sum\limits_{s \in S} {p(s^{\prime } |s,a)} v_{\pi } (s^{\prime } )} \right] \\ \end{aligned}$$

Updating Bellman's expectation equation with the optimal strategy yields the optimal equation as follows:

7 $$V^{ * } (s) = \mathop {\max }\limits_{\pi } V_{\pi } (s) = \mathop {\max }\limits_{\pi } R_{s}^{a} + \gamma \sum {P_{ss^{\prime}}^{a} } V^{ * } (s^{\prime}).$$

### Temporal difference algorithm

The TD algorithm^52^, combining Monte Carlo and dynamic planning methods, uses the classical Bellman formula to iterate until the value function converges. The basic iteration formula is as follows:

8 $$V\left( {s_{t} } \right) = V\left( {s_{t} } \right) + \alpha \left[ {r_{t + 1} { + }\gamma V\left( {s_{t + 1} } \right) - V\left( {s_{t} } \right)} \right].$$

where $r_{t + 1} { + }\gamma V(s_{t + 1} )$ is the objective of TD; $r_{t + 1} { + }\gamma V(s_{t + 1} ) - V(s_{t} )$ is the deviation of TD; $\alpha$ is the learning rate. The procedure to calculate $v(s)$ is given in Algorithm 1.

### Deep learning model

#### Deep neural network

Deep learning^53^ is a type of representation learning that is based on artificial neural networks. Deep neural network structures have greater capacity and exponential representation space, which makes it easier to learn and represent a variety of features with a significantly reduced number of neurons.

The recent success of deep learning relies heavily on massive amounts of training data, flexible models, sufficient computing power, and prior experience to fight against dimensional disasters. Hinton^54^ has proposed a technique combining pre-training and fine-tuning to drastically reduce the time training a multi-layer neural network. Various optimization techniques have emerged to further alleviate the gradient disappearance problem. In particular, an application of a technique known as “Deep Residuals”^55^ can enable more than a hundred network layers.Algorithm 1

**Algorithm 2:**
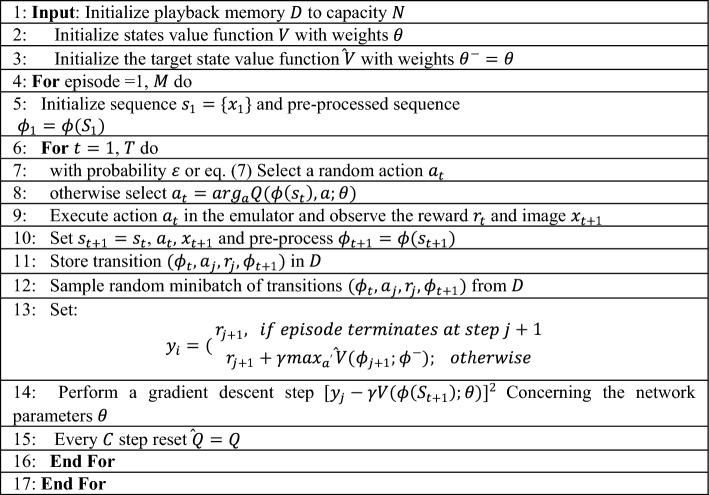
Procedure of DTDN

#### Activation function

The activation function, a central unit in the design of neural networks, gives the ability to learn and adapt for the neurons^56,57^. It incorporates nonlinear factors in the neural network to address the defect of expression ability of the linear model. If the activation function isn’t used, the output of each layer is a linear function of the inputs of the previous layer. No matter how many layers the neural network has, the output is a linear combination of the inputs. Common activation functions include step functions, Sigmoid functions, Tanh functions, and approximate biological neuronal activation functions such as Relu, Leaky-Relu, and Softplus. Because approximate biological neuronal activation functions are better than traditional functions in most network applications, Relu is used in this paper.

#### Optimization function

Optimization function^58^, one of the core problems in neural network training, not only speeds up the solution process but also reduces the influence of hyperparameters on the solution process. Common optimization algorithms used in research applications are the stochastic gradient descent algorithm (SGD), adaptive gradient algorithm (AdaGrad), root mean square prop algorithm (RMSProp), and Adam algorithm.

**Figure 2:**
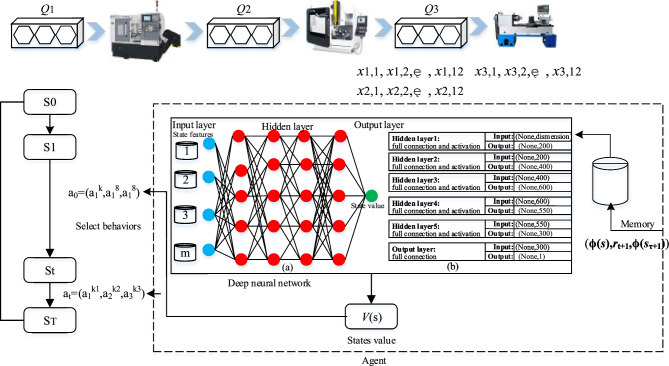
DTDN algorithm running model (3 × 3): Deep neural network model of state perception in agent: (**a**) Deep neural network model of state perception; (**b**) Deep neural network structure.

In this paper, the deep neural network is made up of seven connection layers, which contain one input layer, five implicit layers, and one output layer. Figure 2b gives the structure of the neural network.

### Exploration and exploitation

FDSSP can be classified as a multi-stage decision-making problem with terminals. To balance the allocation of exploration and exploitation of the agent in environmental interactions, a greedy strategy $\varepsilon$ is used as a strategy for selecting behavior. Greedy strategy is the selection of a greedy behavior with probability $1 - \varepsilon$ ($0 < \varepsilon < 1$) and the random selection of any optional behavior with probability $\varepsilon$, where $\varepsilon$ is the exploration factor. Suppose $P(s,a)$ denotes the probability of selecting a behavior at the decision state. The expression is as follows:

9 $$P(s,a) = \left\{ {\begin{array}{*{20}l} {1 - \varepsilon + \frac{\varepsilon }{|A(s)|},} \hfill & {\quad a = a^{ * } (s)} \hfill \\ {\frac{\varepsilon }{|A(s)|},} \hfill & {\quad a \ne a^{ * } (s)} \hfill \\ \end{array} } \right..$$

where $A(s)$ is the set of combinatorial behaviors that are candidates in the state $s$; $|A\left(s\right)|$ is the number of behaviors that can be chosen in the state $s$; ${a}^{*}(s)$ is the greedy behavior of the state. It denotes Eq. (7) as follows:

10 $$a^{ * } (s) = \arg \max \left[ {r_{ss^{\prime}}^{a} + \gamma V(s^{\prime})} \right]$$

where ${r}_{ss{\prime}}^{a}$ is the immediate reward that takes a combination of actions from state $s$ to state ${s}^{,}$.

### Deep temporal difference network model

To briefly describe the implementation process, a workshop visualization ($m = 3,n = 3$) is proposed in Fig. 2. The hexagonal shape represents the jobs. Hexahedra represents waiting for queues of sufficient capacity.

At the start of processing, the scheduling system is in the initial state ${S}_{0}$, i.e., all jobs are in the first waiting queue $Q_{1}$ with all machines free. Then the first machine selects an action $a(k)$ ($1 < k < 8$). A job in the queue $Q_{1}$ is selected for processing while other machines select the action $a(8)$. Whenever any machines complete an operation, the system moves to a new state $S_{t}$. In this state, each machine selects an action to perform. When another operation is completed, the system moves to a new state $S_{{t{ + }1}}$, which gives the agent one reward $r_{t + 1}$. $r_{t + 1}$ can be calculated by the time interval between the two states. Since at each decision moment, each machine simultaneously selects one act to execute. In actuality, the system implements a multidimensional behavior with a combination of m sub-activities at a time in the state $S_{t} (a_{t + 1} = (a_{1} ,a_{2} ,...,a_{m} ))$. When the system reaches the termination state $S_{T}$, it means that all queues are empty and that all jobs have been processed. Hence, a scheduling plan is obtained.

The deep Q-network (DQN) output layer uses several nodes to represent a finite number of discrete action values. However, it cannot cover the exponential multidimensional action space. When the Q-learning online evaluates action values for heterogeneous strategies, it results in over-estimation that optimal value replaces actual interaction values. Hence the method is not directly applied to the multi-dimensional action space problem. Temporal difference learning with the same strategy, state-values indirectly calculating action-values, is proposed to replace Q learning and state values are indirectly calculated for behavior values, which is suitable for selecting multi-dimensional action in Algorithm 2.

## Experiment study

To evaluate the validity of the proposed algorithm, the experiments have been conducted utilizing different test cases in four parts. First of all, according to the standard test set established in Kacem^59^, we use eight small-scale cases to compare with other algorithms^17,32, 60, 61^ in “Small scale FDSSP" section. Then, in “Comparisons with the proposed dispatching rules" section, we compare the proposed DTDN algorithm with the Q-Learning (QL) algorithm (Jiménez^62^) and deep deterministic policy gradient (DDPG) algorithm (Liu^63^) on different performances in Brandimarte^64^. Moreover, for large-scale instances, we designed our test cases, which included 30 FDSSP problems of varying complexity, as shown in Section "Large-scale instances of FDSSP". Last but not least, in "Case Study: production scheduling problem" section, we illustrate in detail the application of our algorithm in a case study of solving the hydraulic cylinder production scheduling problem.

The DTDN algorithm is coded in Python 3.7 language on JetBrains PyCharm Community Edition 2019.2.1 × 64 and runs on Intel Core i9-10900x @ 3.7GHz CPU and 16 GB RAM. First of all, we build FDSSP environment classes, machine classes, and job classes in an object-oriented manner on the RL platform OpenAI Gym. Gym specifies the main member methods of environment classes as a framework, including init, reset, step, render, and close. Then, an agent that executes the algorithm iterates interactively with the environment. The deep neural network model of the agent is implemented with the back-end TensorFlow. The experimental data are shown in Table 5.

**Table 5:** Benchmarks.

Instances	Benchmarks	Source
Case01-05	Kacem01-05	Kacem
Case06-15	Orb01-10	Hurink-data
Case16-20	Mt10c1-xxx	Barnes
Case21-35	01a-15a	ChambersBarnes
Case36-45	MK01-10	Brandimarte

The selection of parameters may affect the quality of the solution, thus general principles can be followed. The discount factor $\gamma$ measures the weight of the subsequent state value on the total return, which is why it generally takes a value close to 1 (i.e.,$\gamma = 0.95$). To facilitate full exploration of the strategy space during the initial phase of the iteration, the $\varepsilon$-greedy strategy sets the initial value of $\varepsilon = 1$ and decays with the discount rate of 0.995. Set the learning rate: $\alpha = 5 \times 10^{ - 4}$; the maximum number of interactions: $MAX - EPISODE = 800$; memory *D* capacity: $N = 6000$; and sample batch: $BATCH - SIZE = 64$. The deep neural network of the agent is shown in Fig. 2b, in which the network parameters adopt a random initialization strategy.

Performance metrics: The relative percentage deviation (RPD) and average relative percentage deviation (ARPD) are described as follows:

$$RPD = \frac{{C_{\max } - LB}}{LB}$$

where $C_{\max }$ are the optimal results of algorithms; $LB$ is the optimal results of the Branch and Bound algorithm. It represents the most ideal solution result and is not possible to achieve.

### Small scale FDSSP

To prove the validity of the solution process in this study, the cases proposed by Kacem. are validated. Where, the number of jobs (*n*), the number of machines (*m*), and each operation of jobs ($O_{ij}$) are represented. For example, $n \times m$ is a case of a set consisting of jobs and machines. The literature with the same case study as this paper is selected for comparison [Zhang^17^ (DACS); Xing et al.^60^ (SM); Moslehi^32^ (PSO); Li et al.^61^ (HTSA)], which ensures the credibility of the comparison results. Meanwhile, each case is run ten times to obtain the combined optimal solution. CPU times of various algorithms are calculated by the “relative ratio” downloaded from https://www.cpubenchmark.net/ (Table 6). The results show that the optimal solution of DTDN and other algorithms are the same as LB, but CPU running time is very significantly different for small-scale problems (Kacem) in Table 7.

**Table 6:** Relative ratio of different computers in studies.

Studies	DACS	SM	PSO	HTSA	DTDN
CPU	2.7 GHz	2.4 GHz	NaN	1.7 GHz	3.7 GHz
CPU mark	7023	228	NaN	132	10,203
Relative ratio	0.6883	0.0223	NaN	0.0129	1.0000

**Table 7:** Results comparison of makespan and CPU time in different methods on Kacem’s test cases.

Prob				B&B		DACS		SM		PSO		HTSA		DTDN	
	m	n	*O*	LB	UB	CM	T(s)	CM	T(s)	CM	T(s)	CM	T(s)	CM	T(s)
Case01	4	5	5	11	NaN	11	0.490	12	2.580	NaN	NaN	11	0.150	11	1.567
Case02	8	8	8	11	NaN	14	2.420	14	39.370	14	NaN	14	3.080	13	2.189
Case03	10	7	7	11	NaN	11	2.100	11	110.000	NaN	NaN	11	2.580	11	3.218
Case04	10	10	10	7	NaN	7	2.560	7	39.740	7	NaN	7	3.120	7	3.189
Case05	15	10	10	11	NaN	11	3.790	11	865.230	11	NaN	11	25.130	11	5.142

As designed in Table 8a, it can be seen that the algorithm progressively generates an optimal production schedule (35). An optimal policy set $\{ \pi^{ * } \} = \{ (6,8,8,8),(2,1,8,8), \ldots ,(8,8,8,1)\}$ is the operation sequence of each job on the machine. Where the number of parentheses in the policy set indicates the combined behavior of the four machines consisting of the behavior number taken in the corresponding state. Where the number in parentheses in the policy set indicates that the four machines in the corresponding state consisting of the behavior number adopt the combined action. At each decision time point, since most of the machine waiting queues are empty or in-process, their feasible action space includes only $a(8)$, which saves computation time. Moreover, the comparison of test results for Problem 2 (Table 8b) shows that the optimal solution of the DTDN algorithm (426) is improved by 4.3% and 2.7% compared to the Nawaz Encore Han (NEH) (445) algorithms and NEH-KK algorithms (438), respectively.

**Table 8:** Design of the test case problems in sets: a (O_5,4_); b (O_6,3_).

Case	1	2	3	4	5	6
1	5/19	4/42	3/85	7/59	3/87	–/42
2	4/46	5/65	2/56	3/68	8/66	–/41
3	5/65	4/12	2/78	6/25	3/53	–/51
4	3/–	5/–	3/–	3/–	7/–	–/–

### Comparisons with the proposed dispatching rules

**Figure 3:**
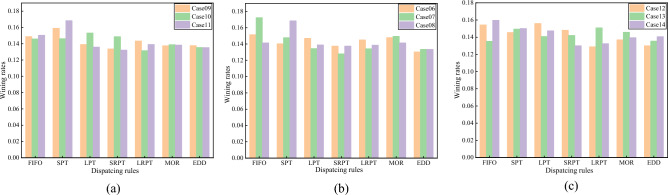
Performance comparison of action space (dispatching rules) under different data cases.

To verify the efficiency and generality of the proposed DTDN, we planned the Brandimarte^64^ data set as our adopted data set. Scores and RPD of the seven dispatching rules on each data case are tallied. As known from Table 9, the proposed DTDN algorithm compared with other algorithms can obtain better solutions, and some of them are already below the upper bound of the original cases. The actions that are used more than 10% are FIFO, SPT, LPT, SRRT, LRPT, MOR, and EDD in Fig. 3. It is known that these actions have a greater contribution to obtaining the optimal solution and thus have a greater utilization value. The frequency distribution of other actions was relatively even, but the performance was not obvious. Therefore, it can be considered to add other heuristic behaviors to the candidate action space, which eliminates some underutilized behaviors to streamline the actions.

**Table 9:** Results comparison of scheduling score and RPD in different methods on Orb data cases.

Prob. (%)	FIFO	SPT	LPT	SRPT	LRPT	MOR	EDD	QL		DDPG		DTDN	
	Score	Score	Score	Score	Score	Score	Score	Score	RPD	Score	RPD	Score	RPD
Case06	7.74e2	7.17e2	7.51e2	7.03e2	7.41e2	7.56e2	6.66e2	9.18e2	1.98e−2	8.75e2	0.1435	8.75e2	1.44e−1
Case07	8.81e2	7.56e2	6.87e2	6.54e2	6.86e2	7.64e2	6.83e2	9.54e2	4.48e−2	8.86e2	0.1284	8.86e2	1.28e−1
Case08	7.15e2	8.52e2	7.03e2	6.96e2	7.01e2	7.15e2	6.75e2	9.18e2	8.96e−2	8.74e2	0.1443	8.51e2	1.75e−1
Case09	7.59e2	8.13e2	7.10e2	6.82e2	7.32e2	7.02e2	7.03e2	9.41e2	6.27e−2	8.88e2	0.1264	8.55e2	1.70e−1
Case10	7.68e2	7.70e2	8.07e2	7.83e2	6.92e2	7.31e2	7.13e2	9.09e2	1.00e−1	8.49e2	0.1701	8.48e2	1.79e−1
Case11	7.59e2	8.49e2	6.85e2	6.67e2	7.02e2	6.98e2	6.82e2	9.49e2	5.35e−2	9.13e2	0.0951	8.49e2	1.78e−1
Case12	8.36e2	7.88e2	8.45e2	8.02e2	6.99e2	7.42e2	7.05e2	9.36e2	6.80e−2	8.48e2	0.1788	8.63e2	1.59e−1
Case13	7.34e2	8.12e2	7.65e2	7.72e2	8.20e2	7.92e2	7.36e2	9.40e2	6.34e−2	8.80e2	0.1369	8.14e2	2.29e−1
Case14	7.86e2	7.40e2	7.26e2	6.41e2	6.53e2	6.87e2	6.93e2	9.38e2	6.63e−2	8.63e2	0.1585	8.49e2	1.78e−1
Case15	7.79e2	7.89e2	7.42e2	7.11e2	7.13e2	7.32e2	6.95e2	9.34e2	7.11e−2	9.75e2	0.1424	8.54e2	1.71e−1

### Large-scale instances of FDSSP

**Figure 4:**
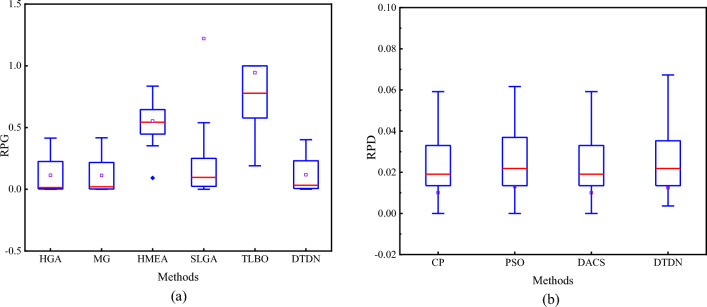
Box plots based on the results in Tables 10 and 11.

In this study, to study the performance of DTDN on large-scale problems, the results of Brandimarte cases are further compared with problem sizes ranging from BC, DP, and BR data cases in a total of 30 data cases. The solution results of the proposed DTDN are compared with Gao et al.^65^ (HGA), Mastrolilli and Gambardella^66^ (MG), Sun et al.^67^ (HMEA), Chen et al.69 (SLGA), and Reddy^68^ [teaching–learning based optimization (TLBO)], which are shown in Table 10 and Fig. 4. The test results show that the proposed DTDN algorithm can find better computational results globally through a large amount of trial and error in the solution procedure. The obtained performance index results are better than traditional optimization methods for different scales of arithmetic cases, demonstrating the validity of the DTDN algorithm for FDSSP.

**Table 10:** Results comparison of scheduling score and RPD in different methods on Orb data cases.

Prob				B&B		HGA		MG		HMEA		DTDN	
	m	n	*O*	LB	UB	Score	RPD	Score	RPD	Score	RPD	Score	RPD
Case16	10	11	1	6.55e2	9.27e2	9.27e2	4.15e−1	9.28e2	4.17e−1	NaN	NaN	9.12e2	3.92e−1
Case17	10	12	1	6.55e2	9.14e2	9.10e2	3.89e−1	9.10e2	3.89e−1	NaN	NaN	9.10e2	3.89e−1
Case18	10	11	1	6.55e2	9.29e2	9.18e2	4.12e−1	9.18e2	4.02e−1	NaN	NaN	9.18e2	4.02e−1
Case19	10	12	1	6.55e2	9.29e2	9.18e2	4.02e−1	9.18e2	4.02e−1	NaN	NaN	9.18e2	4.02e−1
Case20	10	13	1	6.55e2	9.36e2	9.18e2	4.02e−1	9.06e2	3.83e−1	NaN	NaN	9.06e2	3.83e−1
Case21	10	5	1	2.51e3	2.53e3	2.52e3	5.19e−3	2.52e3	4.01e−4	3.83e3	9.22e−2	2.52e3	6.39e−3
Case22	10	5	1	2.23e3	2.24e3	2.23e3	1.36e−3	2.23e3	1.35e−3	3.40e3	5.26e−1	2.35e3	5.66e−2
Case23	10	5	1	2.23e3	2.24e3	2.23e3	4.49e−4	2.23e3	4.49e−4	3.01e3	3.52e−1	2.23e3	2.24e−3
Case24	10	5	1	2.50e3	2.57e3	2.52e3	4.79e−3	2.50e3	0.00e0	3.80e3	5.16e−1	2.52e3	7.59e−3
Case25	10	5	1	2.19e3	2.23e3	2.22e3	1.28e−2	2.22e3	1.23e−2	3.33e3	5.21e−1	2.26e3	1.64e−2
Case26	10	5	1	2.16e3	2.22e3	2.20e3	1.57e−2	2.20e3	1.90e−2	3.11e3	4.37e−1	2.20e3	1.94e−2
Case27	15	8	1	2.19e3	2.41e3	2.31e3	5.49e−2	2.28e3	4.39e−2	3.88e3	7.72e−1	2.32e3	6.13e−2
Case28	15	8	1	2.06e3	2.09e3	2.07e3	5.82e−3	2.07e3	3.88e−3	3.33e3	6.18e−1	2.08e3	9.70e−3
Case29	15	8	1	2.06e3	2.07e3	2.07e3	2.43e−3	2.07e3	2.43e−3	2.98e3	4.47e−1	2.07e3	6.31e−3
Case30	15	8	1	2.18e3	2.36e3	2.32e3	6.29e−2	2.29e3	5.19e−2	4.00e3	8.36e−1	2.35e3	7.94e−2
Case31	15	8	1	2.02e3	2.08e3	2.07e3	0.00e0	2.06e3	2.28e−2	3.17e3	5.70e−1	2.08e3	3.32e−2
Case32	15	8	1	1.97e3	2.05e3	2.03e3	3.10e−2	2.03e3	3.30e−2	3.24e3	6.46e−1	2.04e3	3.35e−2
Case33	20	10	1	2.16e3	2.30e3	2.26e3	4.44e−2	2.26e3	4.59e−2	3.92e3	8.14e−1	2.28e3	5.51e−2
Case34	20	10	1	2.16e3	2.18e3	2.17e3	2.78e−3	2.17e3	2.78e−3	3.45e3	5.96e−1	2.16e3	9.25e−4
Case35	20	10	1	2.16e3	2.17e3	2.17e3	1.85e−3	2.17e3	2.78e−3	3.34e3	5.44e−1	2.17e3	5.09e−3

				LB	UB	HGA		SLGA		TLBO		DTDN	
Case36	10	6	2	3.60e1	4.20e1	4.00e1	1.11e−1	4.00e1	1.11e−1	6.20e1	7.22e−1	4.20e1	1.67e−1
Case37	10	6	3.5	2.40e1	3.20e2	2.60e1	8.33e−2	2.70e1	1.25e−1	4.80e1	1.00e0	3.00e1	2.50e−1
Case38	15	8	3	2.04e2	2.11e2	2.04e2	0.00e0	2.04e2	0.00e0	3.74e2	8.33e−1	2.04e2	0.00e0
Case39	15	8	2	4.80e1	8.10e1	6.00e1	2.50e−1	6.00e1	2.50e−1	1.36e2	2.44e0	6.20e1	2.92e−1
Case40	15	4	1.5	1.68e2	1.86e2	1.72e2	2.38e−2	1.72e2	2.38e−2	2.65e2	5.77e−1	1.72e2	2.38e−2
Case41	10	15	3	3.30e1	8.60e2	5.80e1	7.58e−1	6.90e1	1.10e01	9.40e1	1.85e0	9.00e1	1.73e1
Case42	20	5	3	1.33e2	1.57e2	1.39e2	4.51e−2	1.44e2	8.27e−2	2.46e2	8.50e−1	1.58e2	1.88e−1
Case43	20	10	1.5	5.23e2	NaN	5.23e2	0.00e0	5.23e2	0.00e0	6.23e2	1.91e−1	5.23e2	0.00e0
Case44	20	10	3	2.99e2	3.69e2	3.07e2	2.68e−2	3.20e2	7.02e−2	3.92e2	3.11e−1	3.20e2	7.02e−2
Case45	20	15	3	1.65e2	2.96e2	1.97e2	1.94e−1	2.54e2	5.39e−1	2.75e2	6.67e−1	2.41e2	4.61e−1

### Case study: production scheduling problem

Nourali^8,9^ proposed a useful benchmark of FDSSP, including 40 different data cases. The solution results of the proposed DTDN are compared with Huang et al^69^ [particle swarm optimization (PSO)]; Zhang and Wong^7^ (constraint programming (CP)), and Zhang et al.^17^ [distributed ant colony system (DACS)]. The results are displayed in Table 11 and Fig. 4, and the following conclusions can be summarized. The optimal solutions of this algorithm are all within [LB, UB], indicating that the solutions are valid. The performance of DTDN is close to that of the other three algorithms. The run time from CPU Time is about as long as the other algorithms for small-scale cases, but the large-scale problems are much more efficient than the other method. Lastly, in general, this algorithm is slightly less capable of solving large-scale problems because of the large scheduling state space for large-scale problems, the large learning error using the same network structure, and the need for more iterations and a more optimized network structure to reduce the training error.

**Table 11:** Results Comparison of makespan and RPD in extended Nourali’s test cases.

Prob				B&B		CP		PSO		DACS		DTDN	
	n	m	O	LB	UB	${C}_{max}$	RPD	${C}_{max}$	RPD	${C}_{max}$	RPD	${C}_{max}$	RPD
Ins01	6	2	2	4.16e2	5.23e2	4.23e2	1.68e−2	4.23e2	1.68e−2	4.23e2	1.68e−2	4.23e2	1.68e−2
Ins02	6	2	2	4.56e2	5.12e2	4.64e2	1.75e−2	4.64e2	1.75e−2	4.64e2	1.75e−2	4.64e2	1.75e−2
Ins03	6	2	2	5.10e2	6.17e2	5.17e2	1.37e−2	5.17e2	1.37e−2	5.17e2	1.37e−2	5.17e2	1.37e−2
Ins04	6	2	2	3.78e2	4.42e2	3.89e2	2.91e−2	389e2	2.91e−2	3.89e2	2.91e−2	3.89e2	2.91e−2
Ins05	6	2	2	4.31e2	5.36e2	4.31e2	0.00e0	4.31e2	0.00e0	4.31e2	0.00e0	4.60e2	6.73e−2
Ins06	6	2	3	3.80e2	4.27e2	3.84e2	1.05e−2	3.84e2	1.05e−2	3.84e2	1.05e−2	3.82e2	5.26e−3
Ins07	6	2	3	4.07e2	4.97e2	4.12e2	1.23e−2	4.12e2	1.23e−2	4.12e2	1.23e−2	4.12e2	1.23e−2
Ins08	6	2	3	3.89e2	4.76e2	3.97e2	2.06e−2	3.97e2	2.06e−2	3.97e2	2.06e−2	3.97e2	2.06e−2
Ins09	6	2	3	4.57e2	5.39e2	4.68e2	2.41e−2	4.68e2	2.41e−2	4.68e2	2.41e−2	4.68e2	2.41e−2
Ins10	6	2	3	3.02e2	3.99e2	3.06e2	1.32e−2	3.06e2	1.32e−2	3.06e2	1.32e−2	3.06e2	1.32e−2
Ins11	6	3	2	3.14e2	4.37e2	3.26e2	3.92e−2	3.26e2	3.82e−2	3.26e2	3.82e−2	3.26e2	3.82e−2
Ins12	6	3	2	3.47e2	4.26e2	3.53e2	1.73e−2	3.53e2	1.73e−2	3.53e2	1.73e−2	3.53e2	1.73e−2
Ins13	6	3	2	4.36e2	5.33e2	4.56e2	4.59e−2	4.58e2	4.95e−2	4.56e2	4.59e−2	4.56e2	4.59e−2
Ins14	6	3	2	3.94e2	4.93e2	4.05e2	2.79e−2	4.05e2	2.79e−2	4.05e2	2.79e−2	4.05e2	2.79e−2
Ins15	6	3	2	2.87e2	3.75e2	3.04e2	5.92e−2	3.04e2	5.92e−2	3.04e2	5.92e−2	3.04e2	5.92e−2
Ins16	6	3	3	2.76e2	3.63e2	2.80e2	1.45e−2	2.93e2	6.16e−2	2.80e2	1.45e−2	2.77e2	3.62e−3
Ins17	6	3	3	3.25e2	3.97e2	3.37e2	3.69e−2	3.37e2	3.69e−2	3.37e2	3.69e−2	3.37e2	3.69e−2
Ins18	6	3	3	3.65e2	3.23e2	2.71e2	− 2.57e−1	2.74e2	− 2.49e−1	2.71e2	− 2.58e−1	2.71e2	− 2.58e−1
Ins19	6	3	3	3.46e2	4.33e2	3.54e2	2.31e−2	3.54e2	2.31e−2	3.54e2	2.31e−2	3.54e2	2.31e−2
Ins20	6	3	3	2.98e2	3.77e2	3.09e2	3.69e−2	3.09e2	3.69e−2	3.09e2	3.69e−2	3.08e2	3.36e−2
Ins21	10	2	2	4.22e2	5.87e2	4.38e2	3.79e−2	4.47e2	5.92e−2	4.38e2	3.79e−2	4.36e2	3.32e−2
Ins22	10	2	2	4.76e2	5.98e2	4.97e2	4.41e−2	4.97e2	4.41e−2	4.97e2	4.41e−2	4.97e2	4.41e−2
Ins23	10	2	2	3.43e2	3.95e2	3.54e2	3.21e−2	3.54e2	3.21e−2	3.54e2	3.21e−2	3.54e2	3.21e−2
Ins24	10	2	2	4.32e2	5.33e2	4.46e2	3.24e−2	4.46e2	3.24e−2	4.46e2	3.24e−2	4.46e2	3.24e−2
Ins25	10	2	2	4.87e2	6.23e2	5.07e2	4.11e−2	5.21e2	6.98e−2	5.07e2	4.11e−2	5.07e2	4.11e−2
Ins26	10	2	3	4.21e2	5.47e2	4.48e2	6.41e−2	4.69e2	1.14e−1	4.54e2	7.84e−2	4.68e2	1.12e−2
Ins27	10	2	3	4.67e2	5.98e2	4.86e2	4.07e−2	5.03e2	7.71e−2	4.86e2	4.07e−2	4.86e2	4.07e−2
Ins28	10	2	3	4.02e2	5.88e2	4.22e2	4.98e−2	4.39e2	9.20e−2	4.24e2	5.47e−2	4.22e2	4.98e−2
Ins29	10	2	3	5.03e2	6.98e2	5.25e2	4.37e−2	5.34e2	6.16e−2	5.34e2	6.16e−2	5.25e2	4.37e−2
Ins30	10	2	3	4.75e2	6.21e2	4.79e2	8.42e−3	4.79e2	8.42e−3	4.79e2	8.42e−3	4.79e2	8.42e−3
Ins31	10	3	2	4.25e2	5.22e2	4.25e2	0.00e0	4.27e2	4.71e−3	4.25e2	0.00e0	4.25e2	0.00e0
Ins32	10	3	2	4.24e2	5.17e2	4.34e2	2.36e−2	4.34e2	2.36e−2	4.34e2	2.36e−2	4.34e2	2.36e−2
Ins33	10	3	2	3.35e2	4.73e2	3.68e2	9.85e−2	3.76e2	1.22e−1	3.70e2	1.05e−1	3.70e2	1.05e−2
Ins34	10	3	2	3.74e2	5.27e2	4.01e2	7.22e−2	4.03e2	7.75e−2	4.01e2	7.22e−2	4.22e2	1.28e−2
Ins35	10	3	2	3.86e2	4.87e2	4.09e2	6.00e−2	4.12e2	6.74e−2	4.09e2	6.00e−2	4.10e2	6.22e−2
Ins36	10	3	3	3.93e2	5.07e2	4.15e2	5.60e−2	4.15e2	5.60e−2	4.15e2	5.60e−2	4.15e2	5.60e−2
Ins37	10	3	3	4.13e2	5.37e2	4.52e2	9.44e−2	4.63e2	1.21e−2	4.52e2	9.44e−2	4.52e2	9.44e−2
Ins38	10	3	3	3.87e2	5.89e2	4.19e2	8.27e−2	4.21e2	8.85e−2	4.19e2	8.27e−2	4.19e2	8.27e−2
Ins39	10	3	3	4.97e2	6.27e2	5.21e2	4.83e−2	5.34e2	7.44e−2	5.21e2	4.83e−2	5.21e2	4.83e−2
Ins40	10	3	3	3.69e2	4.79e2	3.86e2	4.61e−2	3.91e2	5.96e−2	3.88e2	5.15e−2	3.84e2	4.07e−2

## Conclusion

The main contribution of this paper is to propose an efficient DTDN method for FDSSP in a flexible shop production environment to minimize makespan. The Q learning in the deep reinforcement learning algorithm DQN is transformed into the temporal differential TD learning with state value. Hence, the deep temporal differential reinforcement learning algorithm is obtained, which is successfully applied to the shop scheduling problem. As shown by experiments, the algorithm can obtain a better solution in a smaller number of iterations compared to simply constructed heuristic or population intelligence algorithms. Because of the introduction of state features, heuristic behaviors, and deep neural networks, the algorithm is highly flexible and dynamic. The advantages of the proposed algorithm include as following:

1. The algorithm can learn and real-time. Since the selection from the input state to the neural network is made by the SCH Algorithm with basic rules. When the neural network is successfully trained, the previous empirical patterns are stored in the network parameters that can make scheduling decisions in real time.
2. The algorithm model is more flexible. The state features, behavior rules, and neural network size can be flexibly modified as needed. The constructive process is closer to the actual scheduling, which is not only applicable to NPFS problems with greater computational complexity but also suitable for solving dynamic scheduling problems from the principle.

### Limitations and future work

Due to the shortcomings of the study, further work can be considered in the following aspects.

1. Scheduling model. Significantly, RL can add and subtract state features to better describe the processing state with minimal redundancy. Searching for more efficient and practical heuristic behaviors can fit and generalize stronger value function generalizer structures. What's more, adding or subtracting candidate behavior sets can be considered to add more highly utilized constructive heuristic behaviors.
2. Algorithm procedure. The DTDN algorithm itself has been proposed after many types of improvements. For example, the DTDN algorithm with priority playback memory for memory sampling priority can improve the efficiency of algorithm iteration.
3. Algorithm application. There is a large development space for the algorithm with the continuous progress of deep neural network theory and the increasing computer computing power. The algorithm can be extended to apply to more complex job shop scheduling problems and other dynamic scheduling problems.

## Data Availability

All data mentioned in the paper are available through Xiao Wang. Email: skwx123@163.com.
